# Successful *Brachyspira hyodysenteriae* Eradication Through a Combined Approach of a Zinc Chelate Treatment and Adapted Management Measures

**DOI:** 10.3390/pathogens15010001

**Published:** 2025-12-19

**Authors:** Frédéric A. C. J. Vangroenweghe

**Affiliations:** 1 BU Swine and Ruminants, Elanco Animal Health Belgium, Generaal Lemanstraat 55/3 (Building D, 1st Floor), 2018 Antwerp, Belgium; vangroenweghe.frederic@telenet.be; Tel.: +32-477-558-562; 2 Unit Porcine Health Management, Department of Internal Medicine–Reproduction–Population Medicine, Faculty of Veterinary Medicine, Ghent University, Salisburylaan 133, 9820 Merelbeke, Belgium

**Keywords:** eradication, *Brachyspira hyodysenteriae*, Zn-chelate, management measures, biosecurity

## Abstract

*Brachyspira hyodysenteriae* is the primary cause of swine dysentery, characterized by bloody to mucoid diarrhea due to mucohaemorhagic colitis in pigs. The disease primarily affects pigs during the growth and finishing stage. The control and prevention of *B. hyodysenteriae* consists of the administration of antimicrobial drugs, in addition to management and adapted feeding strategies. A worldwide re-emergence of the disease has recently been reported with an increasing number of isolates demonstrating decreased susceptibility to several crucially important antimicrobials in the control of swine dysentery. This compromises the possibilities to eradicate *B. hyodysenteriae* from infected pig farms. A novel non-antibiotic zinc chelate has been reported to demonstrate positive effects on fecal quality and consistency, general clinical signs, average daily weight gain, and *B. hyodysenteriae* excretion during and after a 6-day oral treatment. The objective of the present study was to evaluate the zinc chelate (IntraDysovinol^®^ 499 mg/mL; IntraCare) within an eradication schedule with naturally occurring swine dysentery due to *B. hyodysenteriae* resistant to pleuromutilins under field conditions in Belgium. We evaluated a 14-day treatment schedule combined with alternative management measures (including partial depopulation of post-weaning facilities and improved external and internal biosecurity measures) and thorough cleaning and disinfection (including 2% NaOH) of the buildings and the sows from day 7 of treatment onwards. This alternative approach for *B. hyodysenteriae* eradication was evaluated on 18 pig farms over a 5-year period. All enrolled eradication programs were evaluated as successful at least 6–9 months after the finalization of the protocol. In conclusion, the zinc chelate product has an excellent potential for application within an eradication protocol of *B. hyodysenteriae* that are diagnosed as resistant to pleuromutilins. The combined approach of zinc chelate treatment and alternative management measures is demonstrated to be successful in the eradication of farrow-to-wean, farrow-to-finish, and finishing pig farms under field conditions in Belgium.

## 1. Introduction

*Brachyspira hyodysenteriae* (*B. hyodysenteriae*)—a β-haemolytic Gram-negative oxygen-tolerant anaerobic spirochete—is the primary cause of swine dysentery, characterized by bloody to mucoid diarrhea due to mucohaemorhagic colitis in pigs [1]. Swine dysentery primarily affects pigs during the growth and finishing periods, while it is less frequently observed in weaner pigs or sows. The transmission of *B. hyodysenteriae* occurs through the fecal–oral route. The infection is associated with several risk factors such as the introduction of colonized animals (carriers), poor external (quarantine, rodents, wild birds, and other potential reservoirs) and internal biosecurity measures (adequate cleaning and disinfection protocols, mixing of age groups), in addition to nutritional composition such as dietary fiber [2]. The initial onset of the disease is characterized by loss of appetite and mild, yellow to gray-colored diarrhea, further progressing to watery diarrhea with blood, mucus, and pseudomembranes [3]. The disease has been reported in all major pig-rearing countries globally and contributes to significant economic losses, mainly due to growth losses, mortality, increased variation in pig weight, and decreased feed conversion, in the pig production worldwide.

The control and prevention of *B. hyodysenteriae* mainly consists of the administration of antimicrobial drugs, in addition to management and adapted feeding strategies [4]. Currently, no commercial vaccines against *B. hyodysenteriae* are available [1], although some experimental vaccines, such as bacterins, subunit vaccines, and live attenuated strains, have been evaluated [5]. However, EMA has issued a position opinion on the approval of an injectable two-shot inactivated *B. hyodysenteriae* vaccine indicated to protect growing pigs from swine dysentery [6]. Recently, *B. hyodysenteriae* has been reported as a worldwide re-emerging disease with an increasing number of isolates having decreased susceptibility to several crucially important antimicrobials in the control of swine dysentery [7,8,9,10,11,12,13,14,15,16,17,18,19].

Overall, the control and prevention of *B. hyodysenteriae* seems to become more challenging, due to the limited treatment options [2], the lack of effective preventive feeding strategies, and the increased awareness of the reduction in antimicrobial use in animal production [20]. A recent evaluation of different eradication strategies for *B. hyodysenteriae* under Belgian conditions revealed that only 40% of the eradication attempts were finally successful [21]. Three different eradication strategies had been enrolled, namely total depopulation (*n* = 2), partial depopulation combined with antimicrobial treatment (*n* = 6), and only antimicrobial treatment without depopulation (*n* = 2). All farms implemented additional biosecurity measures during and after the eradication program. Based on these results, total depopulation was most successful (100%) followed by partial depopulation combined with an antimicrobial treatment (33%), whereas only antimicrobial treatment without depopulation never succeeded in eradicating *B. hyodysenteriae* (0%) [21]. A 3-week vacancy period for all rooms following cleaning and disinfection, combined with thorough rodent control and additional biosecurity measures, appeared to be the key to a successful eradication. Unfortunately, no clear information on the *B. hyodysenteriae* resistance profile towards pleuromutilins was reported [21].

Due to the reported emergence of antimicrobial resistance, research focused on non-antibiotic alternatives to reduce *B. hyodysenteriae* infections has become more prominent. Adapted feeding strategies, including a high dietary concentration of inulin or citrus extract, have proven to reduce the incidence of swine dysentery due to *B. hyodysenteriae* in grower pigs [22,23]. Among others, zinc has been evaluated as a potential intervention to control *B. hyodysenteriae*. In vitro, the addition of either ZnSO_4_ or CuSO_4_ to the growth medium of *B. hyodysenteriae* caused complete inhibition of hemolytic activity in three culture cycles. Further research revealed that the inhibition of hemolysin was specifically mediated by Zn^2+^ [24]. A comparative study with ZnSO_4_, Zn-methionine, and ZnO only demonstrated the prophylactic effect of high concentrations of in-feed ZnO (2000 ppm or higher) against *B. hyodysenteriae* in a mouse challenge model for swine dysentery [25]. This resulted in the in-feed addition of ZnO in levels of 2500 ppm to reduce the incidence and severity of non-specific cases of post-weaning diarrhea [26]. However, in 2017, the Committee for Medicinal Products of Veterinary Use concluded that the benefits of ZnO for the prevention of diarrhea in pigs do not outweigh the environmental risks of the product [27]. The recent withdrawal of the marketing authorization of ZnO by the European Commission limits the availability of effective alternatives to antimicrobial drugs. Consequently, there is a continuing need for new, effective, non-antibiotic innovations to further improve animal health and welfare and to help reduce economic losses due to *B. hyodysenteriae* infections in pigs.

The chelation of zinc with an inorganic molecule—instead of the covalent binding of zinc to inorganic oxygen—reduces its environmental impact [28]. In addition, previous in vitro (unpublished data) and in vivo studies [28] have demonstrated that a novel organic zinc chelated complex—in relatively low concentrations—is potentially able to reduce the adverse effects due to *B. hyodysenteriae* infections in pigs. The zinc chelate stimulated the in vitro growth of porcine intestinal cells and restored cell viability in the presence of *E. coli* toxins. Moreover, based on a decreased attachment of *E. coli* to cultured intestinal cells in an in vitro cell culture infection model, it is postulated that the zinc chelate might interact with the attachment of *B. hyodysenteriae* outer membrane proteins (OMPs) to the extracellular matrix proteins of the colonic epithelium, reducing the load of *B. hyodysenteriae* in the colon [28]. Another in vivo study demonstrated a positive effect of the zinc chelate on fecal quality and consistency, general clinical signs, and average daily weight gain (ADWG) in *B. hyodysenteriae*-infected animals. Moreover, at the last treatment day, *B. hyodysenteriae* was not detectable by qPCR in most of the treated animals [28]. A previous study has reported no therapeutic effect of 250 ppm zinc chelate in the drinking water for 17 days to pigs inoculated with *B. hyodysenteriae*, which may be due to the nature of the chelating agent used [29]. A field study using this zinc chelate (Intra Dysovinol^®^ 499 mg/mL; IntraCare, Veghel, The Netherlands) demonstrated good clinical efficacy following a 6-day treatment with the recovery of heavily *B. hyodysenteriae*-affected animals [30].

Based on these promising field experiences combined with the urgent need to develop alternative eradication strategies for pig farms infected with a highly resistant *B. hyodysenteriae* strain, the principal investigator in coordination with herd veterinarians and farmers developed and evaluated a practical *B. hyodysenteriae* eradication program using a zinc chelate treatment combined with adapted management measures on 18 *B. hyodysenteriae*-infected pig farms in Belgium.

## 2. Materials and Methods

### 2.1. Selection of the Farms

Belgian pig farms with a positive diagnosis of *B. hyodysenteriae* that had been confirmed resistant to the pleuromutilin antimicrobials (tiamulin, valnemulin) were eligible for the field study. Following first contact, all relevant historic health data as well as diagnostic laboratory information, especially related to *B. hyodysenteriae*, were collected. Subsequently, a farm visit was performed by the principal investigator together with the herd veterinarian to explain the eradication protocol in detail to the farmer. After the first farm visit, the principal investigator prepared a detailed, personalized, farm-specific eradication protocol that was shared with the farm veterinarian and the farmer. Following internal discussion, the plan was refined and finalized during a second farm visit and specific critical control points were addressed and discussed on-farm. A total of 18 pig herds, of which 15 were sow herds and 3 finishing herds, enrolled in this field study (Table 1).

### 2.2. Farm Description and Diagnostic Information About B. hyodysenteriae

A general description of the farms before the implementation of the eradication program is shown in Table 1. The farms were representative for Belgium in terms of size, management practices, biosecurity measures, housing conditions, and nutrition. Mild to severe diarrhea was present in at least one production category (sows, post-weaned piglets, fattening pigs) on all farms. Most of the farms had been suffering from swine dysentery for 2 months to several years prior to the start of the field trial. The eradication protocol was enrolled in the farms included in the field trial between May 2019 and June 2024.

The *B. hyodysenteriae* isolates obtained from the farms were tested for their susceptibility against two antimicrobials commonly used for treatment and eradication of swine dysentery [1], namely tiamulin and valnemulin. Therefore, the agar dilution test as previously described by Vyt and Hommez [19] was used. Isolates were considered resistant if MIC values (µg/mL) were higher than 2 for both tiamulin and valnemulin, respectively [14,31].

### 2.3. Description of the Eradication Strategies

The basic eradication strategy was similar on all farms and included a partial depopulation of the post-weaning facility to obtain at least 3 weeks of sanitary vacancy necessary for thorough cleaning and disinfection, a strict cleaning and disinfection protocol including a 2% NaOH intermediate step between standard cleaning and the final disinfection step of the premises (Table 2) and a specific cleaning and disinfection protocol for the sows (Table 3), a prolonged treatment with an oral zinc chelate product (IntraDysovinol^®^ 499 mg/mL; IntraCare, Veghel, The Netherlands) to eradicate *B. hyodysenteriae* from the colon of infected animals (Table 4), and a series of relevant additional external and internal biosecurity measures to omit re-introduction or internal spread during the eradication process at sow level (Table 5).

### 2.4. Sample Size Calculations

The sample size required to achieve a 90% level of confidence of population freedom of disease was calculated using hypergeometric approximation method [32]. For this calculation, the test’s sensitivity (90%) and specificity (100%), within-herd prevalence (5%; [33,34]), prior confidence of population freedom (75%), probability of introduction (1%), and indicative population size (150–800 sows) were used. The results of this calculation showed that sample size varied between 35 samples for 150 sows and 41 samples for 800 sows to achieve 95% confidence of freedom. Therefore, for sow herds up to 300 sows, we sampled a total of 36 animals pooled per 3 fecal samples (*n* = 12 pools). From 300 to 600 sows, we sampled a total of 39 animals (*n* = 13 pools of 3 fecal samples). In farms with more than 600 sows, we sampled a total of 42 animals (*n* = 14 pools of 3 fecal samples).

### 2.5. Sampling Protocol to Monitor the Infection Status Post-Eradication

Once the entire eradication program had been completed, the farms were monitored for *B. hyodysenteriae* in the feces and for the presence of clinical signs of swine dysentery. Individual fecal samples were collected by the farm veterinarian between 6 and 9 months after the finalization of the eradication program. Additional periodic follow-up visits were performed on a 2-monthly basis by the farm veterinarian to monitor clinical signs for a period of 1 year.

In the sow herds, random samples were collected from 36 (*n* = 12 pools) to 42 (*n* = 14 pools) sows based on the above description concerning specific numbers of fecal samples, whereas in the finishing herds a standard number of 42 (*n* = 14 pools) fattening pigs were sampled. Sampling was mainly performed at stressful moments (weaning, moving production group, change in diet) to increase the likelihood of *B. hyodysenteriae* detection.

The samples were transported cooled in closed plastic recipients to the laboratory (Animal Health Care—Flanders, Torhout, West-Flanders, Belgium) within 2 h after collection.

### 2.6. PCR Analysis of Fecal Samples

Individual fecal samples were pooled for analysis into pools of 3 individual samples [35]. Genomic DNA was extracted from pooled fecal samples. For pooled samples, a total of 3 g of feces—comprising 1 g from each constituent sample—was used for extraction. Each gram of feces was suspended in 5 mL of physiological saline, thoroughly vortexed, and the resulting supernatant was subjected to centrifugation. A 400 µL aliquot of the clarified supernatant was used for the DNA extraction process. Extraction was carried out using the IndiMag Pathogen Kit (Indical Bioscience, Leipzig, Germany), following the manufacturer’s instructions. Subsequent detection of *Brachyspira hyodysenteriae* was performed via real-time PCR using the BactoReal Kit *Brachyspira hyodysenteriae* (Ingenetix, Vienna, Austria), targeting the *nox* gene. Amplification was conducted on an ABI 7500 system (Thermo Fisher Scientific, Waltham, MA, USA), utilizing TaqMan technology with duplex format: the pathogen-specific probe was labeled with FAM™, and the internal positive control (IPC) probe was labeled with Cy5^®^ to monitor for PCR inhibition. The kit incorporates uracil-DNA glycosylase (UNG) and dUTP to mitigate contamination risks.

Thermal cycling parameters were as follows: a 2 min incubation at 50 °C to activate the UNG enzyme, followed by 20 s at 95 °C for initial denaturation; then, 45 amplification cycles, each consisting of 15 s at 95 °C and 1 min at 60 °C, with fluorescence acquisition occurring during the 60 °C step. The assay’s detection limit (LoD95) was established at 18 copies of target nucleic acid per reaction [36].

### 2.7. Bacteriological Analysis and MIC Determination of Fecal Samples

For MIC determination, samples were collected for culture of *B. hyodysenteriae* prior to the onset of the eradication protocol as previously described [21]. Briefly, fecal samples were cultured within 16 h after sampling on selective plates consisting of Trypticase Soy Agar (TSA) (Sigma-Aldrich, St. Louis, MO, USA) supplemented with 5% sheep blood (E&O Laboratories, Bonnybridge, UK), 1% yeast extract (Becton Dickinson, Franklin Lakes, NJ, USA), 6.25 μg/mL vancomycin, 2400 μg/mL spectinomycin, and 6.25 μg/mL colistin (all antimicrobial compounds from Sigma-Aldrich, St. Louis, MO, USA). Plates were anaerobically incubated for 3 days at 41.5 °C and then incubated at 37 °C until 10 days. Suspected colonies were identified by MALDI-TOF MS (Bruker (Billerica, MA, USA), Biotyper). Antimicrobial sensitivity testing was performed by means of an agar dilution method for tiamulin and valnemulin. The plates with the appropriate amounts of antibiotic were prepared as described previously [21]. Tested concentrations ranged from 0.03 µg/mL to 8 µg/mL for both pleuromutilins. The inoculated plates were incubated for 3 days under anaerobic conditions at 37 °C. The MIC was recorded as the lowest concentration at which no distinct hemolysis was observed in the inoculum spot in comparison with the hemolytic effect on the antibiotic-free control plates. The interpretation of the observed MIC values in terms of in vitro efficacy was as follows: tiamulin: susceptible < 1 µg/mL—intermediate 1–4 µg/mL—resistant > 4 µg/mL; valnemulin: susceptible < 1 µg/mL—intermediate 1–4 µg/mL—resistant > 4 µg/mL, as published by Rønne and Szancer (1990) [37] and by Tasker et al. (2004) [38].

## 3. Results

### 3.1. Brachyspira Hyodysenteriae Sensitivity Testing

The antimicrobial sensitivity data revealed MIC values varying between 4 µg/mL and > 8 µg/mL for both tiamulin and valnemulin (Table 6). Therefore, all farms enrolled in the field trial had a moderately to highly resistant *B. hyodysenteriae* strain that did not respond to pleuromutilin treatment anymore.

### 3.2. Eradication Success

All farms (18/18, 100%, CI 95% [82–100%]) enrolled into the field study were demonstrated as *B. hyodysenteriae*-negative at diagnostic sampling 6 to 9 months after the finalization of the eradication protocol (Table 6). In 17 out of the 18 farms, a partial depopulation was implemented at the level of the post-weaning facility, creating a sanitary vacancy of at least 3 weeks to permit the farmer to perform a thorough cleaning and disinfection protocol. On one farrow-to-finish farm, due to the broad extent of the *B. hyodysenteriae* infection within the fattening unit, a total depopulation of the fattening unit was implemented to optimize the eradication success through the reduction in the re-infection risk from ‘infected’ to ‘clean’ fattening pigs during the eradication process.

In all 15 farrow-to-wean or farrow-to-finish herds, an extended treatment with the zinc chelate product (Intra Dysovinol^®^ 499 mg/mL; IntraCare) was implemented for the entire sow population present on the farm. In five of these farms, due to the specific farm building design, post-weaned piglets were also treated with the zinc chelate product to omit the potential risk of the re-introduction of *B. hyodysenteriae* from the piglets back into the sows due to poor internal biosecurity measures. In the three finishing units, specific batches of piglets or finisher pigs were treated with the zinc chelate product to reduce the infection pressure on the site and to omit further spread while the premises were gradually emptied and a thorough cleaning and disinfection procedure was performed.

Detailed information on the typical fecal clinical score, including presence and score of diarrhea (1–4), presence of mucus and blood (0–1), an external and internal biosecurity score (1–10), and the main farm-specific management adaptations that were performed, are given in Table 7.

## 4. Discussion

This study reports a novel approach to *B. hyodysenteriae* eradication using a non-antibiotic zinc chelate product combined with several adapted management measures. Others have reported a variable success rate for the eradication of *B. hyodysenteriae* using different strategies with or without antimicrobial treatment [21]. In a previous study treatment with a zinc chelate in an experimental challenge model with *B. hyodysenteriae* demonstrated no effect on the control or the elimination of *B. hyodysenteriae* [29], which may be due to the nature of the chelating agent used. Unfortunately, no further details on the type of chelating agent were reported [29], which makes comparison with the currently used zinc chelate difficult [28,30].

Critical points identified in previous *B. hyodysenteriae* eradication strategies under local field conditions in Belgium were the presence of antimicrobial resistance, too short (10–14 days) sanitary vacancy period, non-compliance to eradication protocol, or new introduction from outside the farm [21]. Compared with this study, the reasons for success in the current field study were mainly linked to very stringent protocol compliance by the entire farm staff. This was obtained through informative sessions for all staff members to emphasize the importance of the applied management measures prior to the start of the eradication protocol. Another major difference was the length of the sanitary vacancy. In the current study, we advised having at least two production groups (6–8 weeks) distance between the *B. hyodysenteriae*-infected pigs and the piglets born from *B. hyodysenteriae*-eradicated sows in the farm. From an external biosecurity point, a lengthy (6–8 weeks) and thorough quarantine period was implemented for newly introduced breeding gilts into the sow farm.

Farm-adapted *B. hyodysenteriae* eradication strategies were implemented in all farms (*n* = 18) that were enrolled in this field study. The farm-specific strategies were the implementation of a zinc chelate treatment of the sows and/or piglets combined with adapted management measures. Based upon monitoring 6 to 9 months after the finalization of the eradication program, the eradication of *B. hyodysenteriae* was successful in all the infected farms. Indeed, a follow-up period post-eradication of less than 1 year might be too short to conclude that all farms involved in the field study were successfully eradicated for a longer period. However, the principal investigator of the study had regular contact with the farm veterinarians and swine farmers themselves over the period beyond 1 year and none of them reported new clinical signs or outbreaks of disease problems related to the previous *B. hyodysenteriae*. Therefore, based on clinical follow-up, it might be concluded that long-term success was the case, though it has not been documented with further fecal samples for *B. hyodysenteriae* culture or PCR analysis.

The need for an alternative approach towards *B. hyodysenteriae* eradication in this field trial was mainly due to the dramatic increase in antimicrobial resistance of the isolated *B. hyodysenteriae* strains during the last few years [19,39]. Moreover, some of the farms included already performed an unsuccessful eradication attempt using pleuromutilins, before I was contacted to support them with an innovative *B. hyodysenteriae* eradication strategy.

The eradication program enrolled in these *B. hyodysenteriae*-infected farms consisted of a zinc chelate treatment, a thorough cleaning and disinfection protocol of both the premises and sows, a partial depopulation by removal of one or more production batches of piglets or total depopulation of a grow/finishing production group, and the implementation of several relevant farm-specific external and internal biosecurity measures. An important point to mention is that all programs were initiated during the spring or summer period (between the end of April and end of August), as the environmental survival of *B. hyodysenteriae* is much lower during the warm season [3].

The first pillar of the eradication program, a zinc chelate treatment from 7 days prior to the start of the cleaning and disinfection protocol, was essential to remove the pathogen from the intestinal tract of the *B. hyodysenteriae*-infected animals. As previously demonstrated, continuous (24 h) oral treatment with a zinc chelate (IntraDysovinol^®^ 499 mg/L) resulted in *B. hyodysenteriae*-negative fecal samples after 6 days of treatment [28]. A prolonged, off-label treatment under the conditions of the current field trial for at least 14 days could therefore result in a complete clearance of the pathogen from the infected animals. The second period of at least 7 days aimed to protect the animals from potential re-infection during washing and movement from the *B. hyodysenteriae*-contaminated environment to the clean and disinfected environment, which happened from day 7 onwards in the eradication program. In all farrow-to-wean and farrow-to-finish farms, sows were treated with the zinc chelate product to eradicate *B. hyodysenteriae*. In some farms, it was decided to include piglets into this treatment protocol, mainly due to the farm layout, which made it impossible to correctly separate both animal categories in a safe way to omit potential re-introduction from the ‘infected’ post-weaned piglet side to the ‘clean’ sow side of the farm.

The second pillar of the eradication program, thorough cleaning and disinfection, was enrolled as soon as the sows were under treatment for at least 7 days. Besides the standard cleaning and disinfection steps, an additional step applying 2% NaOH (Brenntag, Deerlijk, Belgium) between cleaning and disinfection was introduced. The aim of this supplementary step was to dissolve biofilm layers that might be present on concrete housing elements. These biofilm layers might protect *B. hyodysenteriae* from the subsequent disinfection step and therefore increase the risk of residual infection in the environment. The application of the 2% NaOH should be performed using the correct personal protection material, such as gloves and eye protection. After a 20-to-30 min soaking with this NaOH, the product should be removed through rinsing, prior to the implementation of disinfection. The choice of disinfection product should be based on efficacy against *B. hyodysenteriae* as stated on the label. In our experience, products containing an aldehyde component (formaldehyde, glutaraldehyde, or glyoxal) were suitable for this purpose.

The third pillar of the eradication program, partial or total depopulation of one or more animal production batches (piglets or fattening pigs), was implemented to create a sanitary vacancy of at least 3 weeks between already *B. hyodysenteriae*-negative batches, born from sows that underwent the entire eradication protocol, and still *B. hyodysenteriae*-positive batches, which were considered a potential source of re-infection. Moreover, the sanitary vacancy could optimize the cleaning and disinfection protocol through a prolonged dry empty period of the different compartments. Total depopulation of the fattening unit was only implemented in one farm, mainly due to the nature of the farm layout and complexity, which made only partial depopulation and the related sanitary vacancy difficult to implement correctly.

For the fourth and last pillar of the eradication program, additional external and internal biosecurity measures were advised based on an on-farm visit. These measures were farm-tailored according to the farm layout, specific farm protocols, and motivation of the farmer. The external biosecurity aimed to reduce the risk of the re-introduction of *B. hyodysenteriae* from outside the farm and consisted of a strict entry protocol, including boot cleaning and disinfection, improved quarantine measures for newly introduced breeding gilts, strict control of animal (live and dead) removal protocols, and strict hygiene during manure removal. The internal biosecurity aimed to reduce the risk of spreading *B. hyodysenteriae* from infected compartments on the farm to already eradicated compartments through the clear separation of different animal categories, strict walking lines between animal categories, and strict rodent, bird, and fly control in and around the premises.

Overall, it is important to stress that the high success rate was due to the strict follow-up of the entire eradication program from the initial farm visit, throughout the different preparation steps towards biosecurity measures, including the final execution of the treatment protocol and all the additional management measures. However, since we have no control group only implementing the management measures described in pillars 2 to 4, it is difficult to conclude that the zinc chelate treatment was required to be effective in the control and eradication of *B. hyodysenteriae*. Nevertheless, some of the farms had already performed an earlier eradication attempt using an antimicrobial treatment and the additional management measures described (pillars 2–4) without success. Therefore, if these management measures alone would be sufficient to control or eradicate *B. hyodysenteriae*, then the prior attempts using antimicrobial treatment should also have been successful.

From this field study, even in the presence of antimicrobial resistance to pleuromutilins, the eradication of *B. hyodysenteriae* may still be possible using a non-antibiotic zinc chelate for oral application. However, the eradication program should not solely be based on treatment, since all accompanying measures might be considered as crucial for the success of the current protocol. Although treatment with a zinc chelate product might clear *B. hyodysenteriae* from the colon, potential sources of re-infection on the farm remain present and therefore all presented additional measures should be implemented.

## 5. Conclusions

In conclusion, a combined four-pillar approach—including a zinc chelate treatment, thorough cleaning and disinfection of both the premises and animals, partial depopulation, and additional external and internal biosecurity measures—has been demonstrated to be successful in the eradication of *Brachyspira hyodysenteriae* from farrow-to-wean, farrow-to-finish, and finishing swine farms under field conditions in Belgium.

## Figures and Tables

**Table 1:** Descriptive overview of the different *Brachyspira hyodysenteriae*-infected farms with their detailed management and therapeutic approach towards the eradication of *Brachyspira hyodysenteriae*.

Farm ID	Year	Farm Type	# Sows	Depopulation ^1^	Zn Chelate Treatment Group
A	2019	Farrow-to-finish	225	Partial	Sows
B	2019	Farrow-to-finish	300	Partial	Sows
C	2019	Farrow-to-finish	150	Partial	Sows
D	2019	Farrow-to-finish	500	Total finishing	Piglet/finishers
E	2019	Farrow-to-finish	800	Partial	Sows
F	2019	Farrow-to-finish	500	Partial	Sows
G	2019	Finishing	-	Partial	Piglets
H	2019	Farrow-to-finish	800	Partial	Sows
I	2019	Farrow-to-finish	240	Partial	Sows/piglets ^2^
J	2020	Farrow-to-finish	500	Partial	Sows
K	2020	Finishing	-	Partial	Finishers
L	2020	Farrow-to-finish	400	Partial	Sows/piglets ^2^
M	2020	Farrow-to-wean	500	Partial	Sows/piglets ^2^
N	2020	Finishing	-	Partial	Finishers
O	2020	Farrow-to-finish	250	Partial	Sows
P	2020	Farrow-to-finish	230	Partial	Sows
Q	2024	Farrow-to-finish	800	Partial	Sows/piglets ^2^
R	2024	Farrow-to-finish	100	Partial	Sows/piglets ^2^
S	2024	Farrow-to-finish	300	Partial	Sows/piglets ^2^

^1^ Partial depopulation means the removal of 2–3 batches of post-weaned piglets from the sow farm to create a sanitary vacancy between the piglets born from treated sows and the preceding *Brachyspira hyodysenteriae*-infected post-weaned piglets to be able to run a thorough cleaning and disinfection protocol and omit re-infection through internal spread. ^2^ In case of difficulty to consistently separate sow and piglet category due to specific farm design, both sows and piglets were treated with the Zn chelate product.

**Table 2:** Detailed cleaning and disinfection protocol to sanitize the pig farm environment during the eradication process for *Brachyspira hyodysenteriae*.

Step N°	Description of Specific Action
1	Remove all fecal material manually by shoveling and brushing
2	Soak the surfaces from top to bottom using a detergent solution for at least 1 h
3	Clean the surfaces using a pressure washer
4	Rinse the cleaned surfaces from top to bottom to remove remaining fecal material prior to the next step
5	Apply a 2% NaOH solution (dilute 1 part of 30% NaOH to 14 parts of water) on all non-metal surfaces (concrete, plastic, etc.) and let the solution soak for 20–30 min
6	Rinse the surfaces again from top to bottom with sufficient water to remove the 2% NaOH solution after the required contact period
7	Apply an efficacious disinfectant (containing an aldehyde component) from top to bottom to all surfaces and let the solution work for the recommended contact period (see product information sheet)
8	Rinse the surface from top to bottom to remove the disinfectant solution after the required contact period
9	Dry the surfaces through sufficient ventilation of the compartment for at least 24 h after the final rinsing step

**Table 3:** Detailed cleaning and disinfection protocol to sanitize the sows during the eradication process for *Brachyspira hyodysenteriae*.

Step N°	Description of Specific Action
1	Soak the sows with a suitable shampoo and warm water (30–35 °C)
2	Rinse the sows after the minimal required contact time
3	Specifically focus on thorough cleaning of the hoof section of the limbs
4	Disinfect the entire sow with a suitable disinfectant for topical use in animals
5	Move the cleaned and disinfected sows to a clean section of the gestation room

**Table 4:** Treatment protocol using a zinc chelate product to eradicate *Brachyspira hyodysenteriae* from an infected farm.

Gestating and Lactating Sow Treatment Protocol		
	Determine the water intake of the sows one day prior to the start of the zinc chelate treatment	
	Calculate the daily dose for the sow group with the following formula:	
	S = number of gestating sows BW = estimated/measured average body weight D = dose of zinc chelate product (0.023 mL/kg BW) V = volume of zinc chelate product to be administered daily	**V = S × BW × D**
	Example for 200 sows weighing 245 kg	**1127 mL**
	Dose the product continuously 24/24 for at least 15 days 7 days prior to the start of cleaning and disinfection of the environment and sow washing protocol Extend for another 7 days after the finalization of the above cleaning and disinfection protocol	
	Dose gestating and lactating sows separately due to the major difference in daily water consumption	
Post-weaned piglet treatment protocol		
	Determine the water intake of the post-weaned piglets one day prior to the start of the zinc chelate treatment	
	Calculate the daily dose for the weaned piglet group with the following formula	
	S = number of post-weaned piglets BW = estimated/measured average body weight of the group D = dose of zinc chelate product (0.023 mL/kg BW) V = volume of zinc chelate product to be administered daily	**V = S × BW × D**
	Example for 1000 piglets ranging from 6 to 25 kg (average at 17 kg)	**391 mL**
	Dose the product continuously 24/24 for at least 14 days to clear all potential infection from the colon of the affected piglets	

**Table 5:** Overview of all relevant additional management measures that should be implemented before and during the eradication process for *Brachyspira hyodysenteriae*.

Optimize External Biosecurity Measures to Prevent New Infection Entry	
	Create a hygienic lock with a one-way entry direction including a strict entry protocol
	Farm workers and external visitors **ALL** follow a strict entry protocol Provide clean boots in different sizes
	Thorough boot cleaning and disinfection protocol through installation of disinfection trays for boots using an efficient disinfectant Improved quarantine measures for newly introduced breeding stock
	Loading facilities for reform sow removal with strict one-way protocol
	Removal of dead animals with a strict one-way and end-of-day protocol
	Critical evaluation of manure removal process
Optimize internal biosecurity measures to prevent infection spread from ‘infected’ to ‘clean’ batches during the *Brachyspira hyodysenteriae* eradication	
	Provide separate sets of boots/coveralls for different animal categories **✓** Gestating sows **✓** Lactating sows **✓** Post-weaned piglets **✓** Fattening pigs **✓** Gilt development unit
	Strict walking lines among animal categories
	No return from older to younger age categories with the same clothing and boots
	Strict fly control in the premises Strict bird control in and around the premises
	Strict rodent control in and around the premises

**Table 6:** Pleuromutilin (tiamulin/valnemulin) sensitivity data of isolated *Brachyspira hyodysenteriae* of the different farms prior to the start of the *Brachyspira hyodysenteriae* eradication procedure and outcome of PCR sampling at 6–9 months after end of the *Brachyspira hyodysenteriae* eradication protocol.

Farm ID	Veterinary Practice ID	Year	MIC Tiamulin (µg/mL) *	MIC Valnemulin (µg/mL)	Positive PCR Pool Results 6–9 Months After Eradication
A	1	2019	8	8	0/12
B	2	2019	8	8	0/12
C	3	2019	8	8	0/12
D	4	2019	8	8	0/13
E	5	2019	8	8	0/14
F	6	2019	8	8	0/13
G	7	2019	4	4	0/14
H	7	2019	8	8	0/14
I	8	2019	>8	8	0/13
J	9	2020	8	8	0/13
K	9	2020	8	8	0/14
L	1	2020	>8	8	0/13
M	10	2020	4	4	0/13
N	4	2020	8	8	0/14
O	1	2020	8	8	0/12
P	10	2020	4	4	0/12
Q	3/8	2024	8	8	0/14
S	11	2024	>8	8	0/12

* MIC for tiamulin is reported as >8 µg/mL whenever isolates were still growing at the highest tested MIC of 8 µg/mL.

**Table 7:** Descriptive overview of the fecal clinical score (diarrhea (1–4), mucus (0–1), blood (0–1)) prior to eradication, external and internal biosecurity score (1–10), and main management adaptations related to the eradication on the different *Brachyspira hyodysenteriae*-infected farms.

Farm ID	Fecal Clinical Score			Biosecurity Score		Farm-Specific Management Adaptations
	Diarrhea	Mucus	Blood	External	Internal	
A	3	0	0	7	6	Quarantine, walking lines, rodent control
B	3	0	0	7	7	Walking lines, C&D protocol
C	4	0	0	5	4	General external biosecurity, C&D protocol
D	2	0	0	7	7	Walking lines, sanitary vacancy
E	3	0	0	7	6	C&D protocol, manure evacuation
F	3	0	0	6	6	Sow removal, quarantine, walking lines
G	4	0	0	6	4	Walking lines, manure evaluation
H	3	0	0	5	5	External biosecurity, rodent control
I	3	0	0	7	7	Walking lines, manure evacuation
J	4	0	0	7	6	C&D protocol, walking lines
K	3	0	0	6	6	External biosecurity, C&D protocol
L	3	0	0	8	6	Quarantine, C&D protocol
M	4	0	0	7	7	Sanitary vacancy, rodent control
N	3	0	0	7	6	Walking lines, C&D protocol
O	3	0	0	7	5	Walking lines, sanitary vacancy
P	4	0	0	7	7	Walking lines, rodent control
Q	3	0	0	8	7	Walking lines, rodent control
R	3	0	0	5	4	External biosecurity, walking lines
S	4	0	0	7	6	Walking lines, sanitary vacancy

## Data Availability

The data are unavailable due to the privacy restrictions of the farms and veterinarians involved.
